# Population Dynamics of Patients with Bacterial Resistance in Hospital Environment

**DOI:** 10.1155/2016/1826029

**Published:** 2016-01-24

**Authors:** Leilei Qu, Qiuhui Pan, Xubin Gao, Mingfeng He

**Affiliations:** ^1^School of Mathematical Science, Dalian University of Technology, Dalian 116024, China; ^2^School of Science, Dalian Ocean University, Dalian 116023, China; ^3^School of Innovation and Entrepreneurship, Dalian University of Technology, Dalian 116024, China; ^4^City Institute, Dalian University of Technology, Dalian 116024, China

## Abstract

During the past decades, the increase of antibiotic resistance has become a major concern worldwide. The researchers found that superbugs with new type of resistance genes (NDM-1) have two aspects of transmission characteristics; the first is that the antibiotic resistance genes can horizontally transfer among bacteria, and the other is that the superbugs can spread between humans through direct contact. Based on these two transmission mechanisms, we study the dynamics of population in hospital environment where superbugs exist. In this paper, we build three mathematic models to illustrate the dynamics of patients with bacterial resistance in hospital environment. The models are analyzed using stability theory of differential equations. Positive equilibrium points of the system are investigated and their stability analysis is carried out. Moreover, the numerical simulation of the proposed model is also performed which supports the theoretical findings.

## 1. Introduction

During the past decades, the increase of antibiotic resistance has become a major concern worldwide. Antibiotic resistance is a type of drug resistance where a microorganism is able to survive exposure to an antibiotic. Serious infections caused by bacteria that have become resistant to commonly used antibiotics have become a principle global healthcare problem in the 21st century. Superbugs with the drug-resistant genes are special kinds of bacteria which can be spread by human contact. Drug-resistant genes can be transferred between bacteria in horizontal fashion by conjugation, transduction, or transformation [1, 2]. Therefore a gene for antibiotic resistance which had evolved via natural selection may be shared. In 2010 the August issue of the journal The Lancet: Infectious Diseases, a multinational team reported the emergence and spread of 180 cases of patients infected by bacteria carrying the NDM-1, thus suggesting a widespread dissemination [3]. The horrible problem of NDM-1 is not its ability to attack a person, but the fact that the resistant gene can horizontally transfer to any other bacteria, which can change common bacteria to superbugs.

The World Health Organization announced that the infections caused by superbugs did not respond to conventional treatments, which often induced a long-term illness and greater risk of death. Patients have to spend more time and money because of the bacterial resistance. It will bring huge economic burden and higher risk of death; meanwhile infectious patients will continuously spread superbugs to other people by contact [4]. Therefore, the superbugs bring not only a medical challenge, but also a serious social problem.

The NDM-1 encoding gene is located on different large plasmids that are easily transferable to susceptible bacteria at a high frequency. These plasmids also harbor genes conferring resistance to almost all antibiotics, thus making their rapid dissemination in clinically relevant bacteria a serious threat for therapy [5]. Scientists are afraid that, once resistance genes are combined with certain dangerous bacteria in a multispecies environment, the consequences will be very serious. Due to the superbugs with new drug-resistant genes having just been found, there are mainly experimental studies and testing, and the relevant documents with mathematical models have not been given.

There are some corresponding researches for the dynamic of other resistant bacteria, such as MRSA [6–11] and VRE [10, 12–14]. The literatures about drug resistance under different circumstances, for instance, school, home [15, 16], hospital [8, 9, 11, 13, 17–21], and the community environment, have also attempted to elucidate the mechanisms. Mathematical modeling and simulations are essential approaches to understand biological phenomena. A deterministic mathematical model was developed in papers by D'Agata et al. [8, 17] to characterize the factors contributing to the replacement of hospital-acquired MRSA with CA-MRSA and to quantify the effectiveness of interventions aimed at limiting the spread of CA-MRSA in health care settings. A tremendous variety of models have been formulated recently, mathematically analyzed, and applied to bacterial resistance. Stochastic simulation [9, 13] and agent-based simulation [14, 18–20], combined with hospital environment, are also established. For social environment, researchers are using differential equation models [22–24], stochastic simulation model [25], and neural network simulation model [26] to discuss the dissemination of resistant bacteria.

Opatowski et al. gave a literature review about contribution of mathematical modeling to fight against antibiotic resistance [27]. Temime et al. illustrated the influence of mathematical models in the corresponding research work with example of resistant bacteria and indicated that the importance of mathematical modeling was gradually upgrading [28]. zur Wiesch et al. conducted a review on the generation, evolution, control of resistant bacteria, and so on [29].

The paper is organized as follows. A basic mathematical model is introduced in Section 2; the model depicts the population dynamics of patients with one disease in hospital environment; we analysis the stability properties of the model, and the stability of the disease-free equilibrium and the endemic equilibrium are also discussed. A complex model is given in Section 3, which describes the dynamics of population when two diseases exist in the system. When there are *m* diseases in the hospital environment, the population dynamics is studied in Section 4. Numerical result and parameter analysis are derived in Section 5. The model is analyzed using stability theory of differential equations. Positive equilibrium points of the system are investigated and their stability analysis is carried out. Moreover, the numerical simulation of the proposed model is also performed by using fourth-order Runge-Kutta method which supports the theoretical findings.

## 2. The Basic Model

Patients suffering from a certain disease who enter the hospital are divided into two categories: infected patients and susceptible patients. Here, *I* stands for infected patients, who suffer from the certain disease and are also with bacterial resistance; *S* stands for susceptible patients, who suffer from the certain disease but are without bacterial resistance. The rate of hospital admissions per day is *α*, *p*  (0 < *p* < 1) represents the transfer rate from susceptible patients to infected patients, the cure rate of susceptible patients is *β*, and 1/*β* is the average length of stay. *k*  (0 < *k* < 1) is the resistant strength coefficient; the cure rate of infected patients is reduced to *kβ* because of bacterial resistance. Here, the smaller the value of *k* is, the lower the cure rate of infected patients is, which also means longer average length of stay in hospital. The death rate of patients is *μ*; we assume that bacterial resistance has no effect on mortality but the cure rate and these bacteria are transmitted between patients in hospital via direct contact between patients, through contamination of the institutional environment, or with the inadvertent help of human vectors.

**Figure 1:**
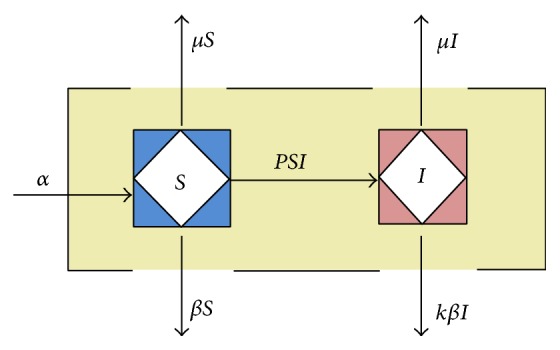
A compartment model of antibiotic resistance in a hospital setting. See text for description and equations.

The model shown in Figure 1 considers the dynamics of patients with bacterial resistance in hospital environment where a single disease exists. The basic system is described by the following set of ordinary differential equations:

(1) S˙=α−pSI−μS−βS,I˙=pSI−μI−kβI.

### 2.1. Steady State Analysis

One of the most important concerns about any infectious disease is its ability to invade a population. An equilibrium point is a point at which variables of a system remain unchanged over time. System (1) possesses the following equilibria.

The model has two types of equilibrium points:

(1) The disease-free equilibrium (DFE) is given by *E*_0_(*S* ^0^, 0) = (*α*/(*μ* + *β*), 0).
(2) The endemic equilibrium is given by *E* ^*∗*^(*S* ^*∗*^, *I* ^*∗*^) = ((*αp* − (*μ* + *β*)(*μ* + *kβ*))/(*μ* + *kβ*)*p*, (*μ* + *kβ*)/*p*).

It is obvious that the endemic equilibrium exists if and only if *αp* > (*μ* + *β*)(*μ* + *kβ*).

### 2.2. Stability of DFE

#### 2.2.1. Local Stability of DFE

To discuss the local stability of equilibrium points we compute the variational matrix of system (1). The signs of the real parts of the eigenvalues of the variational matrix evaluated at a given equilibrium determine its stability. The entries of general variational matrix are given by differentiating the right-hand side of system (1). The matrix is given by

(2) $$\begin{array}{c} J^{*}=\left(\begin{array}{cc} -pI-\mu -\beta & -pS\\ pI & pS-\mu -k\beta \end{array}\right). \end{array}$$

Define

(3) $$\begin{array}{c} R=\frac{\alpha p}{\left(\mu +\beta \right)\left(\mu +k\beta \right)}. \end{array}$$

We get Theorem 1 about the stability of disease-free equilibrium as follows.

**Theorem 1 .**

When *R* < 1, *E*_0_ will be locally asymptotically stable and the endemic equilibrium does not exist.

Otherwise, *E*_0_ is not stable and the endemic equilibrium exists.

**Proof**

It is easy to calculate the characteristic equation of *E*_0_ which is

(4) $$\begin{array}{c} \left(\;\lambda +\mu +\beta \;\right)\left(\;\lambda -pS^{0}+\mu +k\beta \;\right)=0. \end{array}$$

So *λ*_1_ = −*μ* − *β* < 0, and *λ*_2_ = *pS* ^0^ − *μ* − *kβ* = *pα*/(*μ* + *β*) − *μ* − *kβ*.

Considering *S* ^0^ = *α*/(*μ* + *β*) and the defining *R* as that in (3), then when *R* < 1, the eigenvalues has negative real part, which follows that the disease-free equilibrium *E*_0_ is locally asymptotically stable under this condition. Otherwise, the DFE is unstable and the endemic equilibrium exists.

*Biological Meanings*. Here, *R* is the basic reproduction number, which is “the expected number of secondary cases produced, in a completely susceptible population, by a typical infective individual” [30]. The condition *R* < 1 has obviously biological meanings. The drug resistance has the possibility to be extinct in these populations only if the infection rate of drug resistance is small enough. At this moment, the resistant strength coefficient plays a minor role on the dissemination of drug resistance.

#### 2.2.2. Global Stability of Disease-Free Steady State

**Theorem 2 .**

Define *R* as that in (3); then when *R* ≤ 1, the disease-free equilibrium *E*_0_ will be globally asymptotically stable.

**Proof**

To prove the global stability of the disease-free equilibrium, we use the method of Castillo-Chávez et al. [31].

Set *X*_*G*_ = *S*, *Z*_*G*_ = *I*, and rewrite system (1) in the following form:

(5) XG˙=FXG,ZG,ZG˙=GXG,ZG,

in which *G*(*X*_*G*_, 0) = 0.

Represent *E*_0_ by *U*_0*G*_ = (*X*_*G*_^*∗*^, 0). According to the theorem in Castillo-Chávez et al. [31], in order to get the global stability of *E*_0_, system (1) should satisfy three conditions as follows:

(1) *E*_0_ is locally asymptotically stable.
(2) For $\dot{X_{G}}=F(X_{G},0)$, *X*_*G*_^*∗*^ is globally asymptotically stable.
(3) $G(X_{G},Z_{G})=A_{G}Z_{G}-\hat{G}(X_{G},Z_{G}),\;\;\hat{G}(X_{G},Z_{G})\geq 0$, where $A_{G}=D_{Z_{G}}\hat{G}(X_{G}^{*},0)$ is an *M*-matrix.

For system (1), the first condition has been proven in Theorem 1. Since *F*(*X*_*G*_, 0) is a limiting function of $\dot{X_{G}}=F(X_{G},Z_{G})$, that is, lim_*t*→*∞*_ *X*_*G*_ = *X*_*G*_^*∗*^, so the second condition is easy to get.

Now we compute *A*_*G*_ and $\hat{G}(X_{G},Z_{G})$ as follows:

(6) AG=pS0−μ−kβ,G^XG,ZG=pIS0−S=pIαμ+β−S.

For system (1) we get that *S* = *α*/(*μ* + *β* + *pI*), so *S* < *S* ^0^. So $\hat{G}(X_{G},Z_{G})\geq 0$ is always established. Then we conclude that the disease-free equilibrium *E*_0_ is globally stable if *R* < 1. The proof is completed.

### 2.3. Global Stability of the Endemic Steady State

Now let us discuss the stability of *E* ^*∗*^. About the local stability for the disease steady state, we have the following Theorem 3.

**Theorem 3 .**

Define *R* as that in (3). Then when *R* > 1, the endemic equilibrium *E* ^*∗*^ is globally asymptotically stable.

**Proof**

The two equations of system (1) constitute a planar system as follows:

(7) S˙=α−pSI−μS−βS=PS,I,I˙=pSI−μI−kβI=QS,I

in which (*S*, *I*) ∈ *D* = {(*S*, *I*)∣*S* ≥ 0, *I* ≥ 0}. The stability of *E* ^*∗*^ is determined by the value of *p*_1_, *q* in characteristic equation

(8) q∂P∂S∂P∂I∂Q∂S∂Q∂I=−pI−μ−β−pSpIpS−μ−kβ,p1−∂P∂S+∂Q∂I=pI+μ+β−pS+μ+kβ=pI−S+2μ+1+kβ.

When *R* > 1, *q*(*E*_0_) = (*μ* + *β*)(*μ* + *kβ*) − *αp* < 0, the disease-free equilibrium *E*_0_ is unstable. It is easy to verify *q*(*E* ^*∗*^) = *αp* − (*μ* + *β*)(*μ* + *kβ*) > 0 and *p*_1_(*E* ^*∗*^) = *αp*/(*μ* + *kβ*) > 0, so the endemic equilibrium *E* ^*∗*^ is locally asymptotically stable.

Because *D* is a positive invariant set in system (1), to prove *E* ^*∗*^ is global steady in set *D* is equivalent to proving that no periodic orbit of system (1) exists in *D*. We choose the Dulac function *B*(*S*, *I*) = 1/*I*, to evaluate the following expression ∂(*BP*)/∂*S* + ∂(*BQ*)/∂*I* = −*p* − *μ*/*I* − *β*/*I* < 0. So no periodic orbit of system (1) exists and *E* ^*∗*^ is globally asymptotically stable in set *D*.

*Biological Meanings*. When *R* > 1, it means once the patients with drug resistance enter the hospital, the drug resistance will be epidemic. Ultimately the number of patients without drug resistance and patients with drug resistance will be stable in (*μ* + *kβ*)/*p* and

(9) $$\begin{array}{c} \frac{\alpha p-\left(\mu +\beta \right)\left(\mu +k\beta \right)}{\left(\mu +k\beta \right)p}. \end{array}$$

## 3. Bacterial Resistance Spread between Patients with Two Diseases

In this model, we assumed that the patients in the hospital are divided into four compartments:

$S_{1}$ is the population size of patients infected in disease 1 without bacterial resistance at time *t*.
$S_{2}$ is the population size of patients infected in disease 2 without bacterial resistance at time *t*.
$I_{1}$ is the population size of patients infected in disease 1 with bacterial resistance at time *t*.
$I_{2}$ is the population size of patients infected in disease 2 with bacterial resistance at time *t*.

**Figure 2:**
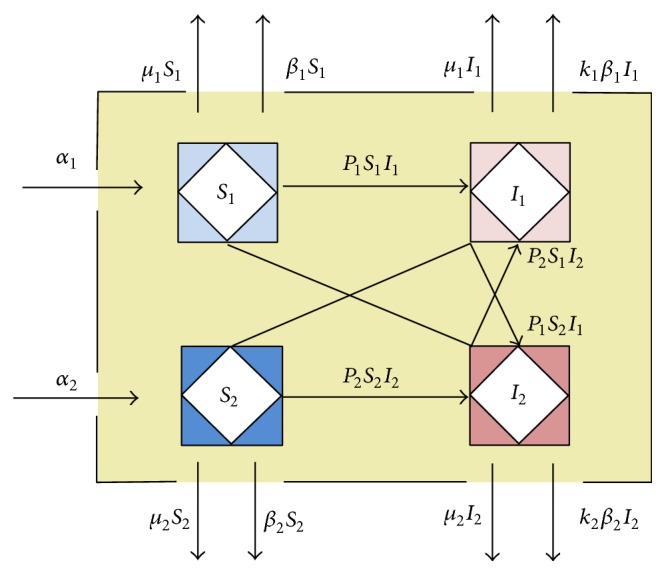
The extended model, in which two diseases exist in hospital setting.

There are two diseases that exist in the hospital environment; disease 1 and disease 2 are not infectious, so we assume that the hospital did not take the isolation precautions. Each kind of patients is divided into infected patients with bacterial resistance *I*_1_, *I*_2_ and susceptible patients without bacterial resistance *S*_1_, *S*_2_. Patients infected with disease *i*  (*i* = 1,2) enter the hospital with the rate *α*_*i*_. The cure rate of susceptible patients is *β*_*i*_, and 1/*β*_*i*_ is the average length of stay. *p*_*i*_  (0 < *p*_*i*_ < 1) represents the transfer rate from susceptible patients to infected patients, *k*_*i*_ is the resistant strength coefficient, and the cure rate of patients *I*_*i*_ is reduced to *k*_*i*_ *β*_*i*_ because of bacterial resistance. The death rate of patients with disease *i*  (*i* = 1,2) is *μ*_*i*_; we also assume that bacterial resistance has no effect on mortality but affects the cure rate. Individuals enter the hospital in one of these states and exit via death or hospital discharge.  Figure 2 shows an expanded form of system (1), in which two diseases exist, called disease 1 and disease 2.

Based on the horizontal of the bacterial resistance between patients, the model is built as follows:

(10) S1˙=α1−P1S1I1−P2S1I2−μ1S1−β1S1,S2˙=α2−P1S2I1−P2S2I2−μ2S2−β2S2,I1˙=P1S1I1+P2S1I2−μ1I1−k1β1I1,I2˙=P1S2I1+P2S2I2−μ2I2−k2β2I2.

### 3.1. Steady State Analysis

System (10) has two possible steady states.

(1) The disease-free equilibrium is given by *E*_0_(*S*_1_^0^, *S*_2_^0^, 0,0) = (*α*_1_/(*μ*_1_ + *β*_1_), *α*_2_/(*μ*_2_ + *β*_2_), 0,0).
(2) The endemic equilibrium is given by *E* ^*∗*^(*S*_1_^*∗*^, *S*_2_^*∗*^, *I*_1_^*∗*^, *I*_2_^*∗*^).

In this section, we analyze the steady states of the model. Define

(11) R01=p1α1μ1+β1μ1+k1β1,R02=p2α2μ2+β2μ2+k2β2.

### 3.2. Stability of DFE

#### 3.2.1. Local Stability of DFE

**Theorem 4 .**

If *S*_1_^0^, *S*_2_^0^, *I*_1_^0^, *I*_2_^0^ ≥ 0, then the solutions are nonnegative and remain bounded in the positive cone of *R* ^4^. If *R*_0_^1^ + *R*_0_^2^ < 1, then the disease-free steady state *E*_0_ is locally asymptotically stable. If *R*_0_^1^ + *R*_0_^2^ > 1, then *E*_0_ is unstable.

**Proof**

It is easy to see that the solutions remain in the positive cone if the initial conditions are in the positive cone. Let *T* = *I*_1_ + *I*_2_ + *S*_1_ + *S*_2_. Then

(12) T.α1+α2−μ1I1−k1β1I1−μ2I2−k2β2I2−μ1S1−β1S1−μ2S2−β2S2=α1+α2−μ1+k1β1I1−μ2+k2β2I2−μ1+β1S1−μ2+β2S2≤α1+α2−min⁡μ1+k1β1,μ2+k2β2,μ1+β1,μ2+β2T.

Thus, the solutions remain bounded in the positive cone of *R* ^4^ and the system induces a global semiflow in the positive cone of *R* ^4^.

To determine the stability of the disease-free steady state *E*_0_, we use the results in van den Driessche and Watmough [32]. Reorder the components of *E*_0_ as *I*_1_^0^ = 0, *I*_2_^0^ = 0, *S*_1_^0^ = *α*_1_/(*μ*_1_ + *β*_1_), and *S*_2_^0^ = *α*_2_/(*μ*_2_ + *β*_2_). Set

(13) FF1F2F3F4=P1S1I1+P2S1I2P1S2I1+P2S2I200,VV−−V+=V1V2V3V4=μ1I1+k1β1I1μ2I2+k2β2I2P1S1I1+P2S1I2+μ1S1+β1S1−α1P1S2I1+P2S2I2+μ2S2+β2S2−α2.

Then

(14) $$\begin{array}{c} F=\left(\begin{array}{cc} \frac{\partial F_{1}}{\partial I_{1}} & \frac{\partial F_{1}}{\partial I_{2}}\\ \frac{\partial F_{2}}{\partial I_{1}} & \frac{\partial F_{2}}{\partial I_{2}} \end{array}\right)=\left(\begin{array}{cc} \frac{p_{1}\alpha_{1}}{\mu_{1}+\beta_{1}} & \frac{p_{2}\alpha_{1}}{\mu_{1}+\beta_{1}}\\ \frac{p_{1}\alpha_{2}}{\mu_{2}+\beta_{2}} & \frac{p_{2}\alpha_{2}}{\mu_{2}+\beta_{2}} \end{array}\right). \end{array}$$

Similarly,

(15) $$\begin{array}{c} V=\left(\begin{array}{cc} \mu_{1}+k_{1}\beta_{1} & 0\\ 0 & \mu_{2}+k_{2}\beta_{2} \end{array}\right). \end{array}$$

Therefore,

(16) FV−1=R01p2α1μ1+k1β1μ2+k2β2p1α2μ1+k1β1μ2+k2β2R02

which implies that the spectral radius of the matrix *FV* ^−1^ is

(17) $$\begin{array}{c} \rho \left(\;FV^{-1}\;\right)=\max\left\{\;0,R_{0}^{1}+R_{0}^{2}\;\right\}. \end{array}$$

If *R*_0_^1^ + *R*_0_^2^ < 1, then *ρ*(*FV* ^−1^) < 1. By Theorem 2 in van den Driessche and Watmough [32], we know that the disease-free steady state *E*_0_ is locally asymptotically stable. *E*_0_ is unstable if *R*_0_^1^ + *R*_0_^2^ > 1.

**Remark 5 .**

The case when *R*_0_^1^ + *R*_0_^2^ < 1 corresponds to the situation that there are no drug resistance strains prevailing in the hospital. Define *R* = *R*_0_^1^ + *R*_0_^2^, where *R* is the reproduction number of system (10).

#### 3.2.2. Global Stability of Disease-Free Steady State

Let

(18) $$\begin{array}{c} R=R_{0}^{1}+R_{0}^{2}. \end{array}$$

**Theorem 6 .**

Define *R* as that in (18); then when *R* ≤ 1, the disease-free equilibrium *E*_0_ will be globally asymptotically stable.

**Proof**

To prove the global stability of the disease-free equilibrium, we use the method of Castillo-Chávez et al.

Set *X*_*G*_ = (*S*_1_, *S*_2_), *Z*_*G*_ = (*R*_1_, *R*_2_), and rewrite system (10) in the following form:

(19) XG˙=FXG,ZG,ZG˙=GXG,ZG,

in which *G*(*X*_*G*_, 0) = 0. Represent *E*_0_ by *U*_0*G*_ = (*X*_*G*_^*∗*^, 0). According to the theorem in Castillo-Chávez et al. [31], in order to get the global stability of *E*_0_, system (10) should satisfy three conditions as follows:

(1) *E*_0_ is locally asymptotically stable.
(2) For $\dot{X_{G}}=F(X_{G},0)$, *X*_*G*_^*∗*^ is globally asymptotically stable.
(3) $G(X_{G},Z_{G})=A_{G}Z_{G}-\hat{G}(X_{G},Z_{G})$, $\hat{G}(X_{G},Z_{G})\geq 0$, where $A_{G}=D_{Z_{G}}\hat{G}(X_{G}^{*},0)$ is an *M*-matrix.

For system (10), the first condition has been proven in Theorem 1. Since *F*(*X*_*G*_, 0) is a limiting function of $\dot{X_{G}}=F(X_{G},Z_{G})$, that is, lim_*t*→*∞*_ *X*_*G*_ = *X*_*G*_^*∗*^, so the second condition is easy to get.

Now we compute *A*_*G*_ and $\hat{G}(X_{G},Z_{G})$ as follows:

(20) AG=p1S10−μ1−k1β1p1S20p1S10p2S20−μ2−k2β2,G^XG,ZG=p1R1+p2R2S10−S1p1R1+p2R2S20−S2.

From system (10) we get that *S*_1_ = *α*_1_/(*p*_1_ *I*_1_ + *p*_2_ *I*_2_ + *μ*_1_ + *β*_1_), so *S*_1_ < *S*_1_^0^. In the same way we get that *S*_2_ < *S*_2_^0^. So $\hat{G}(X_{G},Z_{G})\geq 0$ is always established. Then we conclude that the disease-free equilibrium *E*_0_ is globally stable if *R* ≤ 1. The proof is completed.

### 3.3. Stability of the Endemic Equilibrium

**Proposition 7 .**

Define *R* as that in (18); then the endemic equilibrium *E* ^*∗*^(*S*_1_^*∗*^, *S*_2_^*∗*^, *I*_1_^*∗*^, *I*_2_^*∗*^) is locally asymptotically stable.

We only numerically investigated the system's behavior around the interior feasible equilibrium point *E* ^*∗*^and provide the necessary numerical proof in the next section.

**Theorem 8 .**

No periodic orbit of system (10) exists in

(21) $$\begin{array}{c} \Omega =\left\{\;\left(\;S_{1},S_{2},I_{1},I_{2}\;\right)\;|\;S_{1}\geq 0,S_{2}\geq 0,I_{1}\geq 0,I_{2}\geq 0\;\right\}. \end{array}$$

**Proof**

If a periodic orbit of model (10) in *Ω* exists, its projection onto some two-dimensional subspace of *R* ^4^ should also be periodic. Therefore, we have to investigate if any periodic solution exists or not in an all two-dimensional subspace. There are six different two-dimensional subsystems of (10).

For the subsystem,

(22) S1˙=α1−P1S1I1−P2S1I2−μ1S1−β1S1,S2˙=α2−P1S2I1−P2S2I2−μ2S2−β2S2.

We choose the Dulac function *B*_1_ = 1/*S*_1_ *S*_2_, to evaluate the following expression:

(23) $$\begin{array}{c} \frac{\partial \left(B_{1}\dot{S_{1}}\right)}{\partial S_{1}}+\frac{\partial \left(B_{1}\dot{S_{2}}\right)}{\partial S_{2}}=-\frac{1}{S_{1}S_{2}}\left(\;\frac{\alpha_{1}}{S_{1}}+\frac{\alpha_{2}}{S_{2}}\;\right)<0. \end{array}$$

For the other five subsystems, we choose the Dulac functions

(24) B2=1S1I1,B3=1S1I2,B4=1S2I1,B5=1S2I2,B6=1I1I2.

Similarly

(25) ∂B1S1˙∂S1+∂B1S2˙∂S2=−1S1S2α1S1+α2S2<0,∂B2S1˙∂S1+∂B2I1˙∂I1=−α1S12I1−p2I2I12<0,∂B3S1˙∂S1+∂B3I2˙∂I2=−α1S12I2−p1S2I1S1I22<0,∂B4S2˙∂S2+∂B4I1˙∂I1=−α2S22I1−p2S1I2S1I12<0,∂B5S2˙∂S2+∂B5I2˙∂I2=−α2S22I2−p1I1I22<0,∂B6I1˙∂I1+∂B6I2˙∂I2=−p1S1I12−p1S2I22<0.

Now, using the Bendixson-Dulac negative criterion, no periodic solution in these two dimensions can exist. Therefore, the solution of (10) in *R* ^4^ also cannot oscillate persistently.

## 4. Bacterial Resistance Spreads between Patients with *m* Diseases

There are *m* diseases that exist in the hospital environment; because these diseases are not infectious, we assume that the hospital did not take the isolation precautions. Each kind of patients is divided into infected patients with bacterial resistance *I*_*i*_ and susceptible patients without bacterial resistance *S*_*i*_  (*i* = 1,…, *m*). Patients infected with disease *i*  (*i* = 1,…, *m*) enter the hospital with the rate *α*_*i*_. The cure rate of patients without bacterial resistance is *β*_*i*_, and 1/*β*_*i*_ is the average length of stay. *k*_*i*_ is the resistant strength coefficient; the cure rate of patients *I*_*i*_ is reduced to *k*_*i*_ *β*_*i*_ because of bacterial resistant. The death rate of patients with disease *i*  (*i* = 1,…, *m*) is *μ*_*i*_; we also assume that bacterial resistance has no effect on mortality but the cure rate. Each person may come into contact with an infected type; individuals enter the hospital in one of *S*_*i*_  (*i* = 1,…, *m*) states and exit via death or hospital discharge.

The patients in the hospital are divided into 2*m* compartments, based on the horizontal of the bacterial resistance between patients; the model is built as follows:

(26) Ii˙=∑j=1mpjSiIj−μi+kiβiIi,Si˙=αi−∑j=1mpjSiIj−μi+βiSi,

for *i* = 1,…, *m*, where *x* = (*I*_1_,…, *I*_*m*_, *S*_1_,…, *S*_*m*_). The incidence, *p*_*j*_, depends on individual behavior, which determines the amount of mixing between the different groups.

The DFE for this model is *x*_0_ = (0,…, 0, *S*_1_^0^,…, *S*_*m*_^0^), where *S*_*i*_^0^ = *α*_*i*_/(*μ*_*i*_ + *β*_*i*_).

Linearizing (26) about *x* = *x*_0_ gives

(27) F=Si0pj,V=μi+kiβiδij,

where *δ*_*ij*_ is one if *i* = *j*, but zero otherwise. Thus,

(28) $$\begin{array}{c} FV^{-1}=\left[\;\frac{S_{i}^{0}p_{j}}{\left(\mu_{i}+k_{i}\beta_{i}\right)}\;\right]. \end{array}$$

*F* has rank one, and the basic reproduction number is

(29) $$\begin{array}{c} R_{0}=\overset{m}{\underset{i=1}{\sum }}\frac{S_{i}^{0}p_{i}}{\mu_{i}+k_{i}\beta_{i}}. \end{array}$$

That is, the basic reproduction number of the disease is the sum of the “reproduction numbers” for each group.

## 5. Numerical Result and Parameter Analysis

### 5.1. Numerical Results

The stability and instability of the equilibrium points of the system are studied using the linear stability approach. For further analysis of the steady state of equilibrium points and the parameter effects of *p*_1_ and *p*_2_, we illustrate some key numerical solutions in system (10).

When the parameter values are fixed at *α*_1_ = 4, *α*_2_ = 6, *μ*_1_ = 0.06, *μ*_2_ = 0.06, *p*_1_ = 0.004, *p*_2_ = 0.002, *β*_1_ = 0.2, *β*_2_ = 0.15, *k*_1_ = 0.7, and *k*_2_ = 0.5, we can calculate the equilibrium points as

(30) E0=19.0476,37.5000,0,0E∗=16.9873,32.3503,2.6222,7.4905.

The basic reproduction number is *R* = 1.1436, and as seen in Proposition 7 the equilibrium point *E* ^*∗*^ = (16.9873,32.3503,2.6222,7.4905) is locally asymptotically stable.

The numerical proof of Proposition 7 is as follows.

For the interior equilibrium point *E* ^*∗*^ = (16.9873,32.3503,2.6222,7.4905), the Jacobi matrix is

(31) $$\begin{array}{c} J^{*}=\left[\begin{array}{cccc} -0.2355 & 0 & -0.0679 & -0.0340\\ 0 & -0.1855 & -0.1294 & -0.0647\\ 0.0255 & 0 & -0.0971 & 0.0340\\ 0 & 0.0255 & 0.1294 & -0.0453 \end{array}\right]. \end{array}$$

The eigenvalues of *J* ^*∗*^ are *λ*_1_ = −0.0175, *λ*_2_ = −0.2261, *λ*_3_ = −0.1397, and *λ*_4_ = −0.1801, and all the real parts of the eigenvalues are negative. Hence, the Routh-Hurwitz criteria are satisfied.

Therefore, *E* ^*∗*^ = (16.9873,32.3503,2.6222,7.4905) is locally asymptotically stable.

### 5.2. Role of Parameter *p*_1_ in Model (10)

*p*_1_  (0 < *p*_1_ < 1) represents the conversion rate from susceptible patients to infected patients. Therefore, as *p*_1_ plays a major role in the outcome of the model, we will discuss its effect on the system. The following initial values are used: *S*_1_(0) = 60, *S*_2_(0) = 80, *I*_1_(0) = 4, *I*_2_(0) = 0, and the parameters except *p*_1_ are *α*_1_ = 4, *α*_2_ = 6, *μ*_1_ = 0.06, *μ*_2_ = 0.06, *p*_2_ = 0.001, *β*_1_ = 0.2, *β*_2_ = 0.15, *k*_1_ = 0.7, and *k*_2_ = 0.5.

In order to clearly show population dynamics for each case, two numerical results are given in the following for different values of *p*_1_.

**Figure 3:**
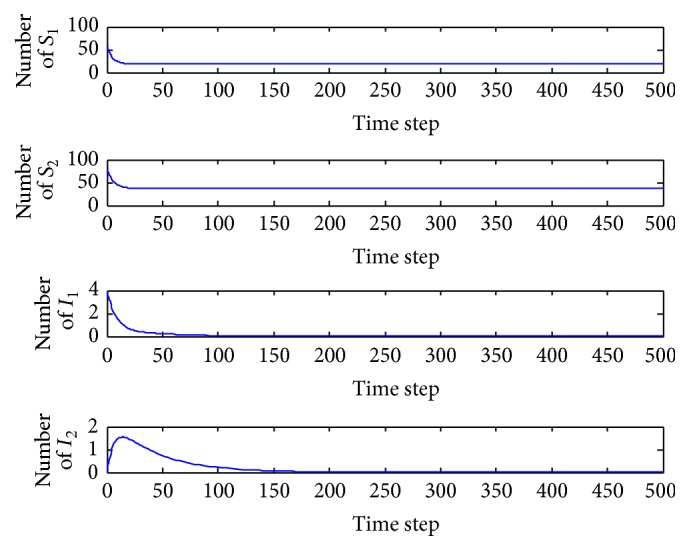
Evolution of the system populations for *p*_1_ = 0.001.

*(a) p*_1_ = 0.001. In this case, the basic reproduction number is *R* = 0.7973 < 1 and so the equilibrium *E*_0_ = (19.0476,37.5000,0, 0) is locally asymptotically stable (Figure 3). Both of the infected patients are extinct, and there are only susceptible patients. In the wide range 0 < *p*_1_ < 0.0028, the behavior of the system is qualitatively the same.

**Figure 4:**
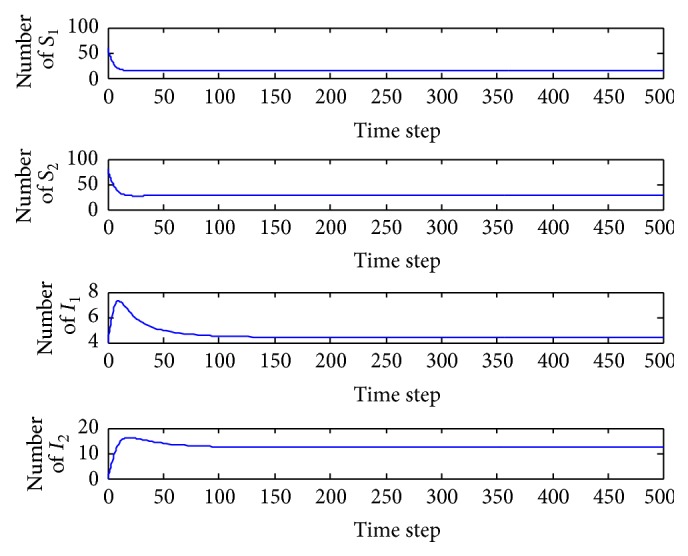
Evolution of the system populations for *p*_1_ = 0.005.

*(b) p*_1_ = 0.005. In this case, the basic reproduction number is *R* = 1.2590 > 1 and so the interior equilibrium *E* ^*∗*^ = (15.5828,29.0286,4.4097,12.3220) is locally asymptotically stable (the proof is provided above). The system tends toward the coexistence equilibrium (Figure 4). When increasing *p*_1_ is from 0.0028 to 1, there is only quantitative change, and the system's qualitative behavior remains the same. Four populations can coexist.

### 5.3. Role of Parameter *p*_2_ in Model (10)

Besides the parameter *p*_1_, we are also concerned about the role of parameter *p*_2_. The initial values are *S*_1_(0) = 60, *S*_2_(0) = 80, *I*_1_(0) = 4, and *I*_2_(0) = 0, and the parameters except *p*_2_ are *α*_1_ = 4, *α*_2_ = 6, *μ*_1_ = 0.06, *μ*_2_ = 0.06, *p*_1_ = 0.004, *β*_1_ = 0.2, *β*_2_ = 0.15, *k*_1_ = 0.7, and *k*_2_ = 0.5.

We describe the influence of *p*_2_ on the system under two different situations.

**Figure 5:**
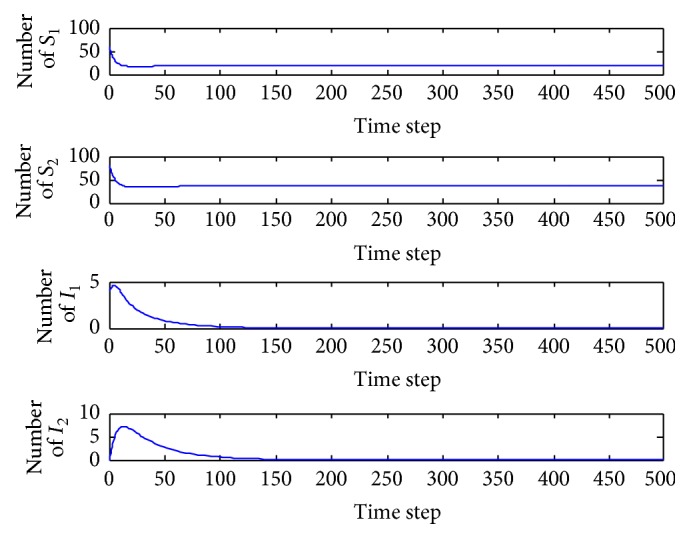
Evolution of the system populations for *p*_2_ = 0.001.

*(a) p*_2_ = 0.001. In this case, the basic reproduction number is *R* = 0.8027 < 1 and so the equilibrium *E*_0_ = (19.0476,37.5000,0, 0) is locally asymptotically stable (Figure 5). Both of the infected patients are extinct, and there are only susceptible patients. In the wide range 0 < *p*_2_ < 0.0016, the behavior of the system is qualitatively the same.

**Figure 6:**
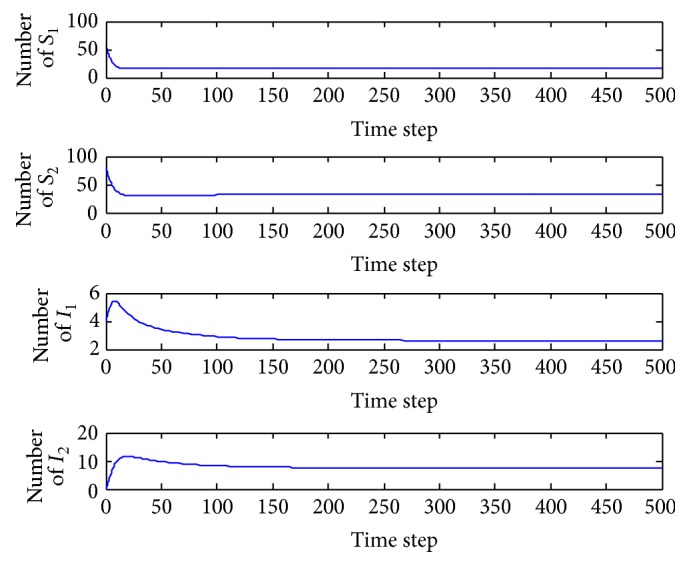
Evolution of the system populations for *p*_2_ = 0.002.

*(b) p*_2_ = 0.002. In this case, the basic reproduction number is *R* = 1.1436 > 1 and so the interior equilibrium *E* ^*∗*^ = (16.9873,32.3503,2.6222,7.4905) is locally asymptotically stable (the proof is provide above). The system tends toward the coexistence equilibrium (Figure 6). When increasing *p*_2_ from 0.0016 to 1, there is only quantitative change, and the system's qualitative behavior remains the same. Four populations can coexist.

### 5.4. Role of Parameter *p*_1_ and *p*_2_ in Model (10)

**Figure 7:**
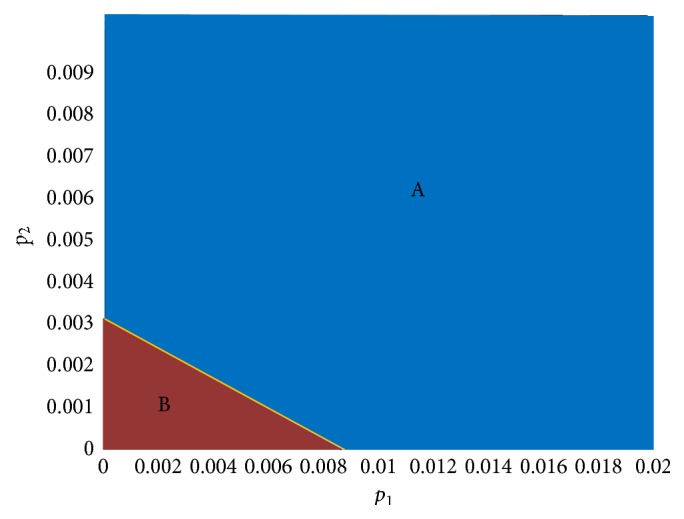
Changing *p*_1_ and *p*_2_ while maintaining the other parameters and the initial values fixed.

In the following section, we discuss the effect of *p*_1_ and *p*_2_ together, while maintaining the other parameters fixed at *α*_1_ = 6, *α*_2_ = 8, *μ*_1_ = 0.06, *μ*_2_ = 0.06, *β*_1_ = 0.2, *β*_2_ = 0.15, *k*_1_ = 0.7, and *k*_2_ = 0.5. The resulting image is shown in Figure 7, in which two different regions were obtained after 500 time steps, which are indicated by different colors.

In region A, four populations coexist. In region B, both of the infected patients are extinct, and there are only susceptible patients. As shown in Figure 7, for a fixed *p*_1_, as the value of *p*_2_ gets smaller, the possibility of coexistence will also be smaller, even becoming impossible.

## 6. Discussion

Two steady states were obtained from the model described in system (10): in the first state, there are only susceptible patients and both of the infected patients are extinct; in the second state, four populations coexist. Among these two cases, only the first is a favorable outcome for humans. As the result shown above, fix one transfer rate, as the value of another transfer rate gets smaller; the possibility of coexistence will also be smaller, even becoming impossible. Meanwhile, the basic production number is determined by the value of transfer coefficients and resistant strength coefficients. Because the resistant strength coefficients are difficult to control, horizontal transfer coefficients are the key parameters that not only can affect the basic reproduction number but also can be controlled by human. There are some papers about how to reduce acquisition of antimicrobial-resistant bacteria [10, 33, 34], such as improving hand hygiene, designing of an efficient sentinel hospital surveillance system, and controlling the connections and number of connections that a given hospital has with other hospitals. In the following work, we will try to add some control measures in the model.
